# Use of Recombinant Human Deoxyribonuclease I in Primary Ciliary Dyskinesia Bronchiectasis—A Real Life Pilot Study

**DOI:** 10.3390/medsci14010133

**Published:** 2026-03-12

**Authors:** Moshe Heching, Liora Slomianksy, Eviatar Naamany, Joel Weinberg, Mordechai R. Kramer

**Affiliations:** 1 Pulmonary Department, Beilinson Hospital, Rabin Medical Center, Petach Tikva 4941492, Israel; 2 Gray Faculty of Medicine and Health Sciences, Tel Aviv University, Tel Aviv 6997801, Israel

**Keywords:** bronchiectasis, primary ciliary dyskinesia, recombinant human deoxyribonuclease, pulmonary exacerbations, mucociliary clearance

## Abstract

*Introduction*: Recombinant human deoxyribonuclease I (rhDNase) cleaves DNA in mucus, facilitating increased mucociliary clearance of purulent sputum. In cystic fibrosis (CF), rhDNase improves pulmonary function and decreases exacerbations. Conversely, rhDNase use in non-CF bronchiectasis (NCFB) patients has not yielded similarly effective results. We explored the safety and feasibility of rhDNase in patients with bronchiectasis due to primary ciliary dyskinesia (PCD). *Methods*: In this real-life pilot study, patients with PCD received rhDNase to treat viscous mucus. We compared pulmonary function tests and pulmonary exacerbations for these patients over six months of use of rhDNase. *Results*: Eight PCD patients with symptomatic bronchiectasis commenced use of rhDNase at variable dosing (ranging from at least twice weekly to a full 2.5 mg dose daily). Over a six-month period, pulmonary function tests, as measured by mean FVC and FEV_1_, remained relatively stable compared to prior to commencing rhDNase. Mean pulmonary exacerbations decreased from 3.1 to 2.3 in the six-month period after commencing rhDNase, as compared to the six-month period prior to rhDNase. *Conclusions*: Use of rhDNase in PCD patients was safe and did not adversely impact lung function or increase pulmonary exacerbations, in contrast to earlier trial results in NCFB patients with heterogeneous etiologies. Further clinical data is required to identify the population of PCD patients who can benefit from rhDNase, as well as the appropriate dosing and timing.

## 1. Introduction

Primary ciliary dyskinesia (PCD) is a rare genetic disorder affecting approximately 1 in 10,000–15,000 individuals, characterized by dysfunction of motile cilia [1]. This dysfunction causes impaired mucociliary clearance, leading to chronic productive cough, recurrent respiratory infections, and progressive bronchiectasis [1].

The pulmonary phenotype of PCD bronchiectasis overlaps with cystic fibrosis (CF). PCD airways exhibit mucus hyperconcentration similar to CF through the formation of hyperconcentrated mucus plaques that obstruct airways and perpetuate chronic inflammation [2]. Like CF, PCD airways are characterized by severe neutrophilic inflammation with elevated concentrations of extracellular DNA, neutrophil elastase, and neutrophil extracellular traps (NETs)—complexes of DNA and cellular proteins released by degraded neutrophils [3,4]. The formation of NETs contributes significantly to increased sputum viscosity and impaired mucociliary clearance, creating a vicious cycle of mucus retention, infection, and progressive lung damage [5].

Deoxyribonuclease I (DNase) is an endogenous enzyme that cleaves DNA into smaller fragments, enabling the clearance of extracellular DNA. Dornase alfa (Pulmozyme^®^, Genentech, Inc. (San Francisco, CA, USA)), an aerosolized recombinant form of human DNase (rhDNase), degrades extracellular DNA in neutrophil-rich mucus and reduces sputum viscosity, facilitating mucociliary clearance of purulent sputum [6]. In CF, rhDNase improves pulmonary function and reduces the frequency of pulmonary exacerbations [7]. The mechanism involves degrading DNA strands derived from neutrophils, thereby reducing the viscoelasticity of airway secretions [6,7]. Accordingly, CF management guidelines recommend the daily inhalation of rhDNase as chronic therapy, particularly for patients with moderate to severe lung disease [8].

In contrast, a 1998 clinical trial evaluating rhDNase in non-CF bronchiectasis (NCFB) found that rhDNase did not yield similar positive results in a heterogeneous population [9]. In fact, there was evidence suggesting potential harm, including a decline in pulmonary function and an increase in pulmonary exacerbations [9]. As a result, guidelines for the treatment of NCFB do not endorse the use of rhDNase in this patient population [10]. However, while the clinical trial enrolled a diverse group of NCFB patients, it did not distinguish the impact of rhDNase across the different etiologies of bronchiectasis. Case reports have suggested that rhDNase may play a beneficial role in patients with PCD-related bronchiectasis [11,12,13]. Given the pathophysiologic similarities between PCD and CF—particularly regarding neutrophilic inflammation, NET formation, and mucus hyperconcentration—there is biological plausibility that PCD patients may respond more favorably to rhDNase than the heterogeneous NCFB population studied in 1998. This study aims to explore the safety and feasibility of rhDNase in patients with bronchiectasis due to PCD.

## 2. Materials and Methods

We conducted a real-life pilot observational study to evaluate the safety and feasibility of rhDNase in patients with PCD-related bronchiectasis, over a period of 12 months from June 2023 to May 2024. Our objective was to monitor PCD patients suffering from viscous mucus who commenced rhDNase to better manage their symptoms. Patients included in the analysis were over 18 years of age, had a confirmed diagnosis of PCD, had symptomatic bronchiectasis (defined by chronicity of symptoms and substantial sputum production), and commenced rhDNase during the study period.

We collected demographic and clinical data, including pulmonary function tests and pulmonary exacerbations, to assess the impact of rhDNase therapy. Pulmonary function tests were compared on the last clinic visit before commencing rhDNase, to the same tests on the first clinic visit after six months had transpired from the beginning of treatment with rhDNase (or the next clinic visit if the patient was experiencing an exacerbation). Pulmonary exacerbations were obtained from the electronic medical record for the six-month periods before and after commencing rhDNase. Pulmonary exacerbations were defined as clinical worsening that necessitated a change in management, specifically the prescription of new oral or intravenous antibiotics due to a worsening of the patient’s clinical status, measured by a decline in pulmonary function and an increase in cough, dyspnea, or sputum amount or viscosity. A schematic flow diagram of the methodology for the study is presented in Figure S1.

## 3. Results

During the study period, eight PCD patients with symptomatic bronchiectasis commenced use of rhDNase to better manage their symptoms. Patients used rhDNase up to once daily, guided by the amount and viscosity of their sputum, in some cases reducing the dosage to less than a full dose (2.5 mg/2.5 mL once daily) as well as the frequency of use. Patients continued their other medications, including hypertonic saline, inhaled antibiotics, and macrolides (if any), as well as airway clearance therapy.

As shown in Table 1, the patients had a mean age of 60 years (±8.8, range 42–73), 63% male, and a mean forced expiratory volume in the first second (FEV_1_) of 46%. Five patients were of European descent and three of Middle Eastern descent. Six of eight patients were chronically infected with *Pseudomonas aeruginosa*. The use of rhDNase varied, with four patients using rhDNase daily at full dose, two patients using a half-dose (1.25 mg/1.25 mL) of rhDNase daily, one patient using a full dose of rhDNase every other day, and one patient using a full dose of rhDNase intermittently based on symptoms, at least twice weekly. Importantly, only one in eight patients discontinued use of rhDNase during the observation period, and no patients were lost to follow-up during the study.

Pulmonary function tests, as measured by mean forced vital capacity (FVC) and FEV_1_, remained relatively stable compared to baseline. There was a small but not clinically significant increase in mean FVC. Importantly, we observed a reduction in pulmonary exacerbations, with the mean number of pulmonary exacerbations decreasing over the six-month periods before and after commencing rhDNase from 3.1 to 2.3, respectively. No formal quality of life questionnaire or assessment was conducted; however, patients did informally report that rhDNase improved their airway clearance and reduced the sputum burden.

## 4. Discussion

Our results provide preliminary support for the safety and feasibility of rhDNase in PCD patients with symptomatic bronchiectasis suffering from viscous mucus. Significantly, in contrast to the 1998 study findings for NCFB with heterogeneous etiologies, we did not observe a trend towards reduction in pulmonary function, nor an increase in pulmonary exacerbations. Rather, we observed stability in pulmonary function tests and a reduction in pulmonary exacerbations, implying that in the PCD patient population, the use of rhDNase can be safe and therefore should not be categorically discouraged.

While the mean FEV_1_ for each patient remained unchanged, on an individual basis, some patients did experience a decrease in FEV_1_, particularly those using a full daily dose. This decrease could be related to the rhDNase treatment, although other factors may also have played a role, including decreased adherence to airway clearance and inhalation regimens. Of note, in the pivotal phase 3 trial for rhDNase in CF patients, approximately 7% of patients experienced a >10% decrease in FEV_1_ during the clinical trial [14]. More significantly, none of the individual patients in our cohort experienced an increase in exacerbations; exacerbations for each patient either decreased or remained unchanged.

Chronic inflammation in bronchiectasis leads to the activation and overstimulation of neutrophils, disrupting their normal life cycle [15]. Neutrophil extracellular traps (NETs) are complexes of DNA and cellular proteins, formed through the expulsion into the airways of chromatin and cellular DNA from degraded neutrophils [4]. The formation of NETs results in the accumulation of extracellular DNA debris in the airways, which becomes incorporated into airway mucus, thereby increasing its viscoelasticity and impeding mucociliary clearance, leading to airway obstruction [5].

Dornase alfa (Pulmozyme^®^), a bioengineered equivalent of DNase, degrades NETs by hydrolyzing the DNA within them, breaking DNA into smaller polymers, and thereby reducing sputum viscosity and improving airway clearance [16]. In CF patients, this leads to decreased sputum viscosity, improved pulmonary function tests, and reduced pulmonary exacerbations [7]. The pivotal phase 3 trial for the approval of dornase alpha demonstrated both an improvement in pulmonary function tests and a reduction in pulmonary exacerbations over a six-month period [14].

Studies on PCD-related bronchiectasis have highlighted significant similarities to CF, particularly regarding the presence of NETs and their role in contributing to sputum viscosity [3]. Both PCD and CF airways are characterized by severe neutrophilic inflammation, with high concentrations of extracellular DNA and proteases such as neutrophil elastase, key contributors to increased sputum viscosity and impaired mucociliary clearance [3].

In PCD, sputum neutrophils are activated and dysfunctional, with evidence of increased neutrophil elastase activity, paralleling the inflammatory milieu seen in CF [17]. Elevated DNA concentrations and similar biophysical sputum properties suggest that NETs play a role in PCD comparable to CF [18]. The overlap in inflammatory markers, including neutrophil elastase, and the lack of significant differences in sputum viscosity between CF and PCD, further support this similarity [19].

Because NETs contribute to airway epithelium damage and increase mucus viscosity, rhDNase can potentially facilitate the degradation of NETs in PCD [18]. Furthermore, the highly viscous sputum can inhibit the penetration of inhaled antibiotics in bronchiectasis, and a reduction in DNA in the sputum through rhDNase may increase the bioactivity of inhaled antibiotics in PCD patients [20].

Our results stand in contrast with the 1998 study on rhDNase in NCFB. The trial followed patients for six months and, in contrast to the trials in CF patients, demonstrated a decrease in pulmonary function in the treatment arm (both FEV_1_ and FVC), while the placebo arm remained relatively stable [9]. The authors did note, however, that the difference in pulmonary function (−3%) was likely not clinically significant, and importantly, no differences were observed in quality of life [9].

In contrast to our study, the cohort in the NCFB study included participants with bronchiectasis with varied or idiopathic etiologies. As such, the conclusions of the 1998 study on rhDNase may not be directly applicable to PCD, given the similarities between CF and PCD in terms of sputum mucin concentrations, as compared to NCFB [2]. The contrasting results between our PCD cohort and the 1998 NCFB trial may be explained by differences in airway mucus pathophysiology across bronchiectasis etiologies. While direct head-to-head comparisons across all bronchiectasis etiologies are limited, available data indicate that CF and PCD exhibit similarly elevated sputum mucin concentrations (approximately 6500–7000 µg/mL total sputum mucin concentration) as compared to heterogeneous NCFB (approximately 4000–5000 µg/mL) [2,21]. These quantitative differences in mucin concentration correlate with sputum viscosity and disease severity [2]. A recent study has shown that viscoelastic properties and inflammation markers in the sputum of PCD patients are comparable to those of CF patients treated with CFTR modulators, supporting the treatment of mucus dysfunction in PCD with therapies targeted at the mucus pathophysiology found in CF [22]. As noted above, case reports have demonstrated the efficacy of rhDNase in children and adolescents with PCD [11,12,13].

Moreover, in the NCFB study, nearly 50% of the patient cohort had a history of smoking [4], implying that the etiology of bronchiectasis may have been smoking related for a substantial number of the participants, and patients with COPD have substantially lower mucin concentrations, as compared to CF and PCD [2].

Additionally, the NCFB trial used rhDNase twice daily; although both once and twice daily regimens are approved, clinical practice supports a twice daily regimen in the context of worsening lung function and pulmonary exacerbations; in a more stable patient population, a once daily regimen is preferred [23]. With the twice-daily regimen, sputum can become too diluted, and excessive dilution of mucus can lead to sputum becoming too liquid and difficult to expectorate. Nevertheless, despite this dosing difference between studies, we believe the more fundamental explanation for the divergent outcomes lies in the underlying disease pathophysiology. Importantly, even our patients receiving 2.5 mg once daily—the same per-day dose as one of the arms in the pivotal CF trials—did not experience the decline in lung function or increased exacerbations observed in the NCFB trial cohort. This suggests that disease-specific factors, particularly the pathophysiologic similarities between PCD and CF regarding neutrophilic inflammation and mucin concentration, are likely more important determinants of rhDNase response. The biological plausibility for this study’s findings lies in the shared pathophysiology between PCD and CF. The heterogeneous nature of the NCFB trial population, which included varied and idiopathic etiologies, may explain why the results are not directly applicable to PCD patients who share the neutrophil-rich, DNA-laden, viscous sputum characteristics of CF. Nevertheless, these observations are preliminary based on a pilot study, and we acknowledge that definitive conclusions require prospective comparative studies directly measuring mucin concentrations and sputum properties across well-defined bronchiectasis etiologies.

This study has various limitations, including the small sample size, variable dosing, single center and open-label design. Given the small sample size, the findings should be interpreted with caution and may not be generalizable. The variable dosing regimen across patients precludes dose–response analysis and limits our ability to determine optimal dosing strategies. While this individualized approach is consistent with standard clinical practice for inhaled therapies in bronchiectasis, future larger studies should prospectively evaluate optimal dosing strategies for PCD patients. The demographic distribution (age/gender) may introduce sampling bias. No pattern by age or gender was observed, but the study is underpowered to detect such differences. Larger cohort studies and multi-center trials are necessary to confirm these results and better understand the potential benefits of rhDNase in patients with PCD-related bronchiectasis. PCD patients in our cohort had substantially viscous sputum, which may help guide clinicians in selecting appropriate candidates for treatment.

## 5. Conclusions

The use of rhDNase in PCD patients did not adversely impact lung function or increase pulmonary exacerbations, in contrast to earlier trial results in NCFB with heterogeneous etiologies. The airway mucin concentration of PCD is more similar to CF than to idiopathic bronchiectasis, which may explain the contrasting results from NCFB. When used judiciously, rhDNase can be safe for use in PCD patients and warrants further investigation. Further clinical studies are needed to identify the subset of PCD patients who would benefit from rhDNase, as well as to determine optimal dosing and timing. We look forward to multi-center trials in PCD patients to guide future practice and guidelines regarding rhDNase use in PCD bronchiectasis.

## Figures and Tables

**Table 1:** Demographic and clinical data of patients prior to commencing rhDNase compared to six months post commencing rhDNase. PEx = number of pulmonary exacerbations. NA = not applicable, patient discontinued treatment after four months of treatment. nNO = nasal nitric oxide. VM = high-speed video microscopy. EM = transmission electron microscopy.

					FVC			FEV_1_			
Age	Gender	Dosage	Pre FVC	Post FVC	Change %	Pre FEV_1_	Post FEV_1_	Change %	Pre PEx	Post PEx	PCD Diagnosis
62	F	1 daily	90	86	−4	75	68	−7	2	1	Genetics, nNO
63	M	Intermittent	45	45	0	50	49	−1	3	3	nNO, VM
58	M	1 daily	71	76	5	56	51	−6	4	3	nNO, VM
58	F	Every other day	38	46	8	31	34	3	NA	NA	VM, EM
73	F	1 daily	32	43	11	26	28	2	3	2	nNO, VM
60	M	1/2 daily	54	54	−1	34	36	1	4	4	nNO, VM
42	M	1 daily	47	48	1	48	50	2	4	2	Genetics, nNO
66	M	1/2 daily	74	77	3	52	56	4	2	1	Genetics, nNO
Mean curent study			55	58	2.9	46	46	0.0	3.1	2.3	
Mean from O’Donnell et al. (Chest 1998) [9]					−3.4			−3.6			

The red color is intended to show that there was a decline (negatie number).

## Data Availability

The data presented in this study are available on request from the corresponding author due to patient confidentiality and regulatory restrictions.

## Supplementary Materials

The following supporting information can be downloaded at: https://www.mdpi.com/article/10.3390/medsci14010133/s1. Figure S1: Methodology Flow Diagram.

## Abbreviations

CF	cystic fibrosis
DNase	deoxyribonuclease I
FEV_1_	forced expiratory volume in the first second
FVC	forced vital capacity
NCFB	non-CF bronchiectasis
NETs	neutrophil extracellular traps
PCD	primary ciliary dyskinesia
rhDNase	recombinant human deoxyribonuclease I
