# Supplementary figures and images for: Use of Recombinant Human Deoxyribonuclease I in Primary Ciliary Dyskinesia Bronchiectasis—A Real Life Pilot Study
Source: Med Sci (Basel). 2026 Mar 12;14(1):133. doi: 10.3390/medsci14010133 (PMC13027975; doi:10.3390/medsci14010133)

Figure S1. Methodology Flow Diagram

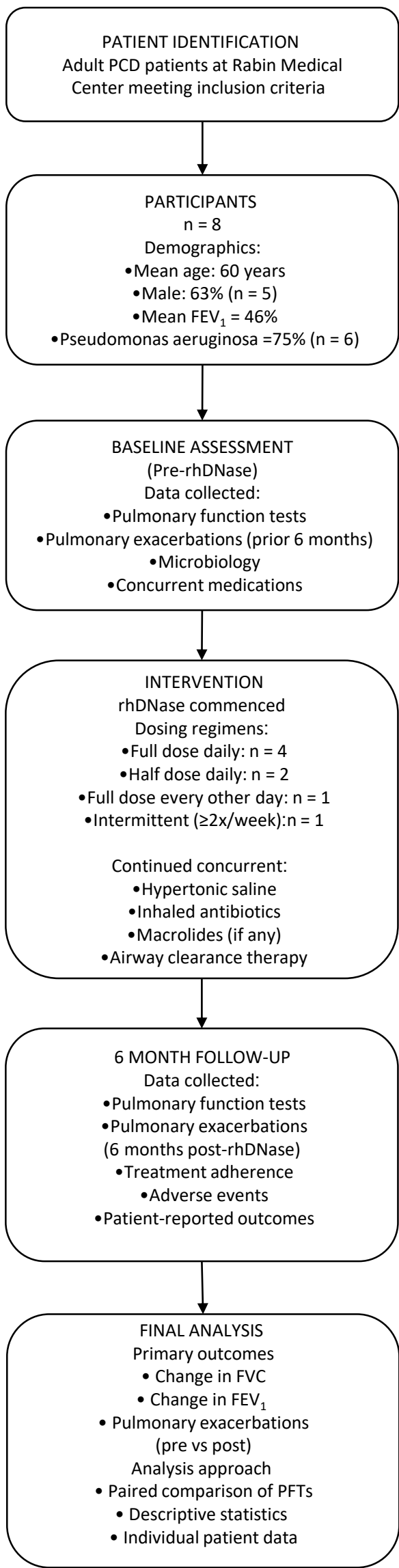

Supplement: Supplementary file 1 [file medsci-14-00133-s001.zip › medsci-4134065-supplementary.pdf]
